# Supplementary material for: Comparative genomics provides new insights into the diversity, physiology, and sexuality of the only industrially exploited tremellomycete: Phaffia rhodozyma
Source: BMC Genomics. 2016 Nov 9;17:901. doi: 10.1186/s12864-016-3244-7 (PMC5103461; doi:10.1186/s12864-016-3244-7)
Supplement: Additional file 6: — List of orphan genes with links to PFAM (related to Additional file 1: Table S1). (ZIP 1428 kb) [file 12864_2016_3244_MOESM6_ESM.zip › PFAM_UNIQUE/G04558_P.html]

#                                                                            --- full sequence --- -------------- this domain -------------   hmm coord   ali coord   env coord
  
# target name        accession   tlen query name           accession   qlen   E-value  score  bias   #  of  c-Evalue  i-Evalue  score  bias  from    to  from    to  from    to  acc description of target
  
#------------------- ---------- ----- -------------------- ---------- ----- --------- ------ ----- --- --- --------- --------- ------ ----- ----- ----- ----- ----- ----- ----- ---- ---------------------
  
G04558\_P             -            577 DAZAP2               PF11029.3    137      0.67    9.1   8.9   1   2   2.9e-05     0.058   12.5   3.7    37   104   151   228   120   232 0.70 - DAZ associated protein 2 (DAZAP2)  
G04558\_P             -            577 DAZAP2               PF11029.3    137      0.67    9.1   8.9   2   2       2.8   5.6e+03   -3.6   0.2    63    71   454   462   447   483 0.68 - DAZ associated protein 2 (DAZAP2)  
G04558\_P             -            577 p47\_phox\_C           PF08944.6     58       2.9    6.8   8.8   1   2   0.00052      0.78    8.7   3.4     7    39   194   225   189   226 0.86 - NADPH oxidase subunit p47Phox, C terminal domain  
G04558\_P             -            577 p47\_phox\_C           PF08944.6     58       2.9    6.8   8.8   2   2      0.87   1.3e+03   -1.6   0.2     1    17   258   272   258   283 0.53 - NADPH oxidase subunit p47Phox, C terminal domain  
G04558\_P             -            577 Ste5                 PF11610.3     30     0.074   11.3   0.6   1   2   7.7e-05      0.46    8.7   0.1     6    18   314   326   310   336 0.78 - Scaffold protein Ste5-Fus5 binding region  
G04558\_P             -            577 Ste5                 PF11610.3     30     0.074   11.3   0.6   2   2     0.076   4.5e+02   -0.8   0.1     7    13   465   471   463   476 0.78 - Scaffold protein Ste5-Fus5 binding region
